# Morphology control of nickel nanoparticles prepared in situ within silica aerogels produced by novel ambient pressure drying

**DOI:** 10.1038/s41598-020-68510-4

**Published:** 2020-07-16

**Authors:** Jialu Lu, Jiabin Wang, Khalil T. Hassan, Alina Talmantaite, Zhengguang Xiao, Michael R. C. Hunt, Lidija Šiller

**Affiliations:** 1 School of Engineering, Newcastle University, Newcastle upon Tyne, NE1 7RU UK; 2 Centre for Materials Physics, Department of Physics, Durham University, Durham, DH13LE UK

**Keywords:** Engineering, Materials science

## Abstract

Silica aerogels are low density solids with high surface area and high porosity which are ideal supports for catalyst materials. The main challenge in aerogel production is the drying process, which must remove liquid from the pores of the wet gel while maintaining the solid network. In this work, the synthesis of silica aerogels and nickel-doped silica aerogels by a low energy budget process is demonstrated. Silica aerogels are produced by ambient drying using ammonium bicarbonate, rather than a conventional low surface tension solvent. Heating dissociates the ammonium bicarbonate, so generating CO_2_ and NH_3_ within the pores of the wet gel which prevents pore collapse during drying. Nickel-doped aerogels were produced by reducing nickel ions within pre-synthesised silica aerogels. The morphology of the resulting nickel particles—spheres, wires and chains—could be controlled through an appropriate choice of synthesis conditions. Materials were characterized using nitrogen adsorption/desorption isotherms, scanning electron microscopy, Fourier-transform infrared spectroscopy, thermogravimetric analysis and X-ray diffraction. The surface area of undoped aerogel is found to increase with the concentration of ammonium bicarbonate salts from 360 to 530 m^2^ g^−1^, and that of nickel-doped silica aerogel varies from 240 to 310 m^2^ g^−1^ with nickel doping conditions.

## Introduction

Silica aerogels are ultra-low-density solids with the vast majority (typically > 90%^1^) of their volume made up of voids consisting of mesopores and macropores. This unique structure leads to a variety of extraordinary properties such as extremely high specific surface area, low dielectric constant and low thermal conductivity, opening up potential uses including in catalysis, adsorption for pollution remediation, thermal super-insulation and in drug delivery systems^2–8^. Practical application of aerogels has, however, been limited by high materials costs and laborious methods for drying. These issues have been solved only recently by the development of a low-cost ambient pressure drying (APD) approach^9^ for aerogel production by Han and co-workers. In this method the reaction of sodium bicarbonate with HCl, generated from tetramethylchlorosilane (TMCS), was used to generate pore-supporting carbon dioxide within the wet gel during the drying process, so avoiding the need for low surface tension solvents.

Nickel catalysts supported on silica have been employed for the reforming of carbon dioxide and methane to produce synthesis gas (syngas—typically a mixture of H_2_, CO and CO_2_)^10^ which are important intermediates for the production of synthetic natural gas and methanol. When immobilised within silica aerogels, nickel nanoparticles (NiNPs) and nanowires (NiNWs) have been shown to catalyse the CO_2_ hydration reaction (CHR) which has significant potential for carbon capture, storage and utilisation (CCSU) to mitigate anthropogenic climate change^11^. Similarly, CO_2_ reforming of CH_4_ has been demonstrated with nickel doped alumina aerogel catalysts^2^. However, for the CHR to occur in nickel nanostructure embedded aerogels, it is a requirement that the aerogel is hydrophilic, since the CHR mechanism involves the formation of hydroxyl groups on the nickel surface from water. These are then converted to bicarbonate by the nucleophilic attack of CO_2(aq)_, which is displaced by H_2_O^12^. Unless water can efficiently penetrate the aerogel to reach the embedded nickel no significant CHR can occur.

It is well known that morphology can have a significant impact on the physical^13^ and chemical^13–15^ behaviour of nanoparticles. For example, Hassan et al*.* demonstrated that pre-prepared NiNPs immobilised on silica aerogels were more active for CHR than nickel nanowires (NiNWs) embedded in the same aerogel matrix^11^. However, in our previous work the synthesis of nickel-doped aerogels involved the use of pre-prepared commercially-sourced NiNPs and NiNWs which were physically dispersed in the sol, just prior to gelation^11,16^. In this work we report the *morphologically controlled* synthesis of nanoparticles and nanowires achieved in situ through reducing nickel chloride absorbed in the aerogel. By control of the synthesis conditions we demonstrate that spherical nanoparticles, needles or chain-like wires supported by a silica aerogel can be produced, enabling particle shape to be tailored for specific applications. This simple new method can, in principle, be applied to any hydrophilic aerogel.

In addition, in this work, we have modified the APD approach of Han et al*.*^9^ by employing the thermal decomposition of ammonium bicarbonate to generate pore-supporting gases (NH_3_ and CO_2_) within the wet gel. This approach has significant advantages over the previous method since it eliminates the initial “pre-drying” stage and no salts are produced, removing the need for a washing step to produce a pure silica aerogel.

## Experimental

### Preparation of materials

**Figure 1:**
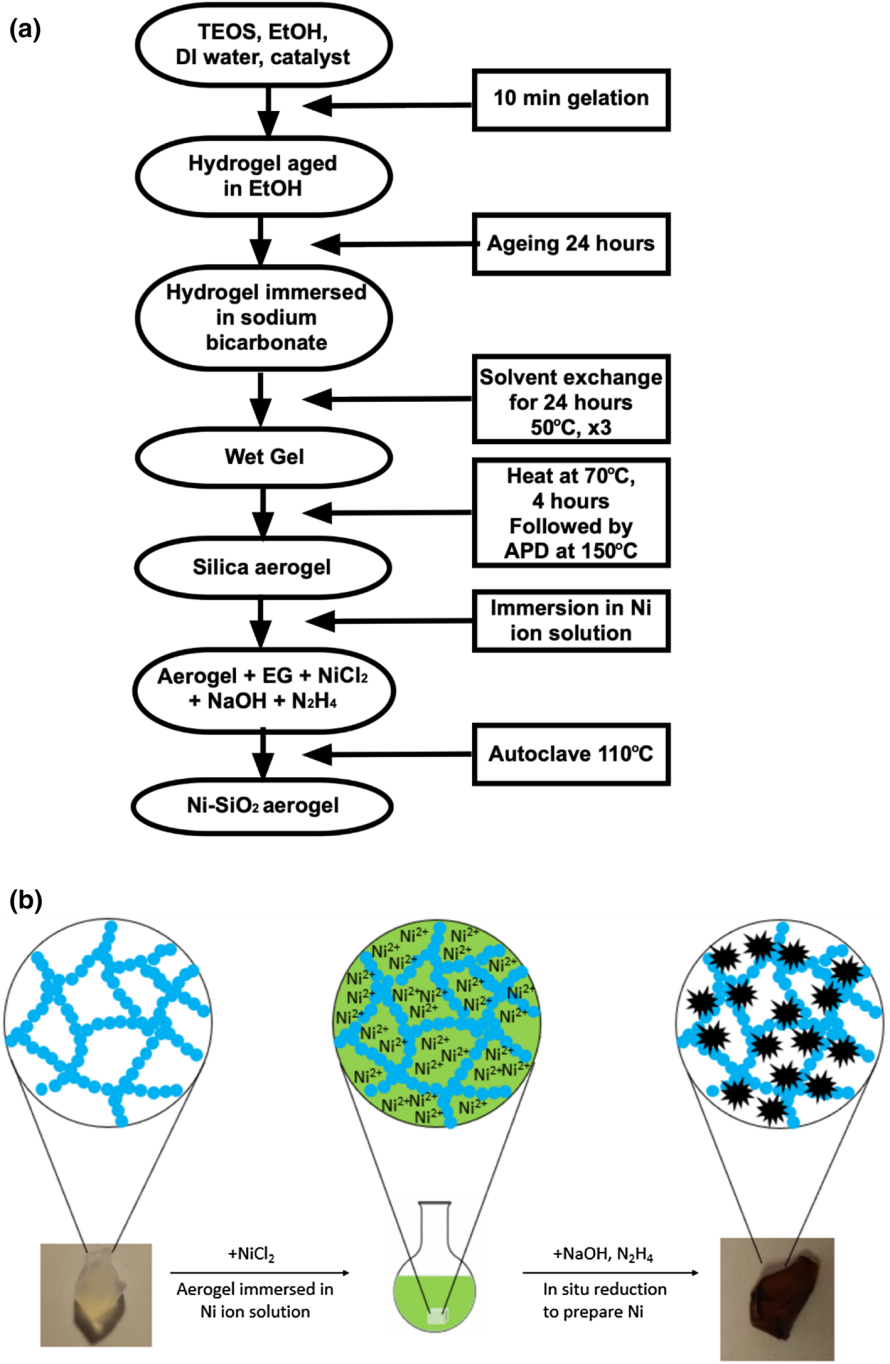
(**a**) Flow diagram illustrating the aerogel preparation process; (**b**) Schematic illustration of the in situ synthesis of Ni within the aerogel, corresponding to the last two steps in (**a**).

Tetraethyl orthosilicate (TEOS, 98%), ammonium hydroxide (28–30%), ammonium fluoride (98%), ammonium bicarbonate (99.5%), ethanol (99.8%), hydrazine (60%), nickel chloride (99%), sodium hydroxide (99%), ethylene glycol (99.8%) were all purchased from Sigma-Aldrich (UK) and used without any further purification. Silica hydrogels were prepared using TEOS as the precursor, following the first five steps of the flow chart shown in Fig. 1a. In brief, 4.4 ml TEOS was mixed with 20 ml ethanol and 6.4 ml ultra-high purity (UHP) water (18 MΩ cm^−1^), followed by 0.4 ml of basic catalyst to promote gelation. The catalyst used for this experiment has a mole ratio of ammonium hydroxide: ammonium fluoride: H_2_O of 8:1:111. The resulting mixture was poured into a casting mould and held at room temperature for 10 min. The wet gel was then removed from the mould and aged in ethanol for 24 h. After aging, the hydrogels were immersed ammonium bicarbonate solution from 3 to 25% and are named according to the scheme indicated in Table 1. For the solvent exchange step 500 ml of ammonium bicarbonate solution was used, changed three times every 24 h and the sample and solvent heated to 50 °C. Following solvent exchange, the wet gels were heated to 70 °C for 4 h while still immersed in the ammonium bicarbonate solution. The solution was then removed and the gel dried at 150 °C under ambient conditions.

**Table 1:** Ammonium bicarbonate concentrations used to prepare silica aerogel samples and resulting physical properties.

Sample	NH_4_HCO_3_ (wt%)	BET surface area (m^2^ g^−1^)	Average pore diameter (nm)	Pore volume (cm^3^ g^−1^)	Porosity (%)
DSA15	3	360	2.7	0.72	96.9
DSA50	10	370	3.1	0.73	96.9
DSA75	15	420	4.6	0.83	96.8
DSA100	20	460	5.3	0.86	97.0
DSA125	25	530	6.1	0.87	95.9

To produce nickel-doped silica aerogels a similar approach to that used by Wu and Chen^17^ for the production of nanoparticle suspensions was employed, which will be referred to as the ‘polyol process’^18^ . 0.1 g nickel chloride was dissolved in 6 ml ethylene glycol followed by the addition of 2 ml of NaOH solution at different concentrations, as outlined in Table 2 along with the sample naming convention. Hydrazine, N_2_H_4_ (300 μl), was then added to the resultant solution. Samples of dried silica aerogel produced using 20 wt% ammonium bicarbonate (DSA100) were soaked in the nickel ion solution until the color of the gels had changed uniformly. The reduction of nickel ions to metallic nickel by hydrazine occurs via the following scheme^17^:

$$2{\rm Ni}^{2+}+{\rm N}_{2}{\rm H}_{4}+4{\rm OH}^{-}\to 2{\rm Ni}+{\rm N}_{2}+4{\rm H}_{2}{\rm O}$$

The polyol process has the distinct advantage that it produces pure Ni nanostructures without the requirement of an inert atmosphere.

**Table 2:** NaOH concentrations used in the preparation of nickel-doped aerogels with the resulting physical properties and nickel nanocrystal morphology.

Sample	NaOH concentration (M)	BET surface area (m^2^ g^−1^)	Average pore diameter (nm)	Pore volume (cm^3^ g^−1^)	Morphology of nickel nanocrystals
Ni1	0.5	300	4.6	0.89	Sphere
Ni2	1	310	4.5	0.90	Needle
Ni3	2	240	5.4	0.91	Wire

The gels, impregnated with nickel solution, were subsequently placed in an autoclave heated to 110 °C for 4 h. After autoclaving, the nickel-doped aerogels were washed first in ethanol and then UHP water for three cycles. A flow chart outlining the complete process is presented in Fig. 1a and a schematic of the nickel doping process in Fig. 1b.

### Materials characterisation

Materials were characterized at Newcastle University, with the exception of Scanning Electron Microscopy which was performed at the G.J. Russell Microscopy Facility, University of Durham. X-ray diffraction (XRD) analysis was undertaken using a PANalytical X'Pert Pro Multipurpose Diffractometer using Cu Kα X-rays. Samples were mounted on a low-background silicon substrate and diffraction scans covered a 2*θ* range of 10°–90°. A ThermoScientific Surfer system was used to determine specific surface area and pore size distributions in the samples. The specific surface area was found by measuring the adsorption of gaseous N_2_ using a Brunaer–Emmett–Teller (BET) analysis and has an error of ± 24 m^2^ g^−1^, whilst pore volumes have an error of ± 0.02 cm^3^ g^−1^. Pore distributions were obtained from the nitrogen adsorption/desorption isotherms by the Barrett–Joyner–Halenda (BJH) method and the uncertainty in the average pore size is ± 0.1 nm. Before N_2_ adsorption, all samples were degassed at 200 °C. Fourier transform infrared (FT-IR) spectroscopy was measured in the range of 500–4,000 cm^−1^ with a Varian 800 FT-IR spectrometer, to confirm the surface chemical structure of the wet and dried gel. The thermal stability of the aerogel samples was examined via thermogravimetric analysis using a DynTHERM analyser. A FEI Helios Nanolab 600, operated in SEM mode at a beam energy of 10 keV was used to determine the microstructure of the undoped aerogel samples, which were coated with the minimum thickness of gold required to eliminate the effects of sample charging. The microstructure of nickel doped samples was measured with a FEI XL30 ESEM-FEG scanning electron microscope operated at a beam energy of 20 keV and equipped with an energy dispersive X-ray (EDX) analyser.

## Results and discussion

XRD scans obtained from undoped silica aerogels present a broad and strong diffraction peak which can be observed between 2*θ* angles of 20° and 30° and is characteristic of amorphous silica^16,19^, with no significant differences observed between the samples (see Figure S1, Supplementary Material). No evidence is observed for any other crystalline phase present within the samples, suggesting that the ammonium bicarbonate fully decomposes at all concentrations used.

**Figure 2:**
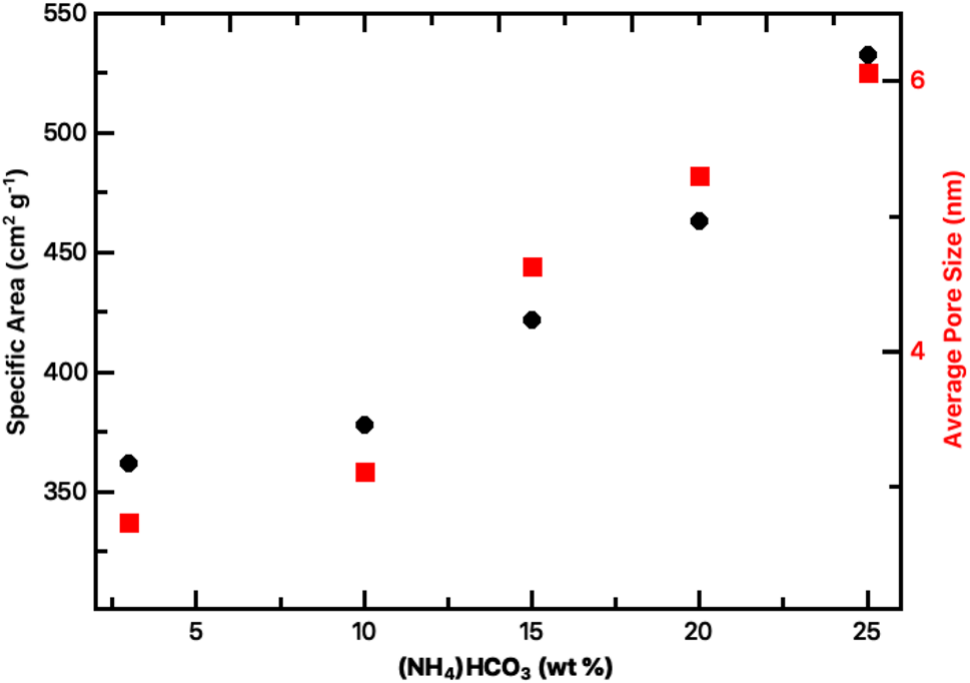
Variation of average pore size and specific surface area with ammonium bicarbonate concentration in undoped silica aerogels.

Nitrogen adsorption/desorption isotherms were used to determine the specific surface area, average pore diameters and pore volume of each of the silica aerogel samples (Table 1). Porosity was estimated from the mass of sample (measured to and accuracy of 0.1 μg) using a density of amorphous silica of 2.196 g cm^−3^ and the relation *P* = *V*_*p*_/*V*_*t*_, where *P* is porosity, *V*_*p*_ the pore volume and *V*_*t*_ the total volume of the sample. As shown in Table 1 porosity of our samples is ~ 97%. All adsorption–desorption isotherm showed typical Type IV behavior, indicating that the aerogels produced in this study are mesoporous. Specific surface area is observed to increase monotonically with ammonium bicarbonate concentration from 360 to 530 m^2^ g^−1^, with a linear variation above 10 wt%, demonstrated in Fig. 2. An almost identical variation in average pore size from 2.7 to 6.1 nm is also observed. Based on the mechanism of aerogel formation through the generation of pore-supporting gases within the wet gel, proposed by Han et al*.*^9^, these results indicate that increasing the volume of pore-supporting gas within the wet gel leads directly to a larger pore size in the resultant aerogel and a resultant increase in specific surface area. The linear relationship between the concentration of ammonium bicarbonate and average pore size/specific surface area above 10 wt.% concentration indicates that it is straightforward to tailor the aerogel pores for specific applications through an appropriate choice of precursor concentration.

**Figure 3:**
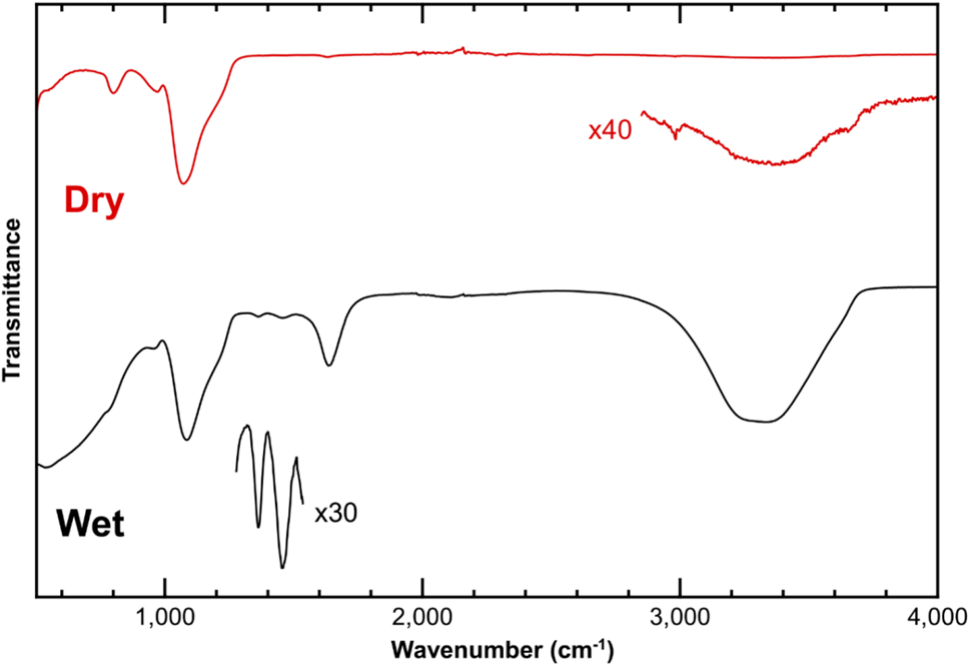
FT-IR spectra of a wet gel (black curve) and dried silica aerogel (red curve). The magnified region of the wet gel spectrum shows IR modes associated with the HCO_3_^−^ and NH_4_^+^ ions**,** whilst that of the dry aerogel shows the –OH stretching region of water.

Typical FT-IR spectra from a wet gel and a dried silica aerogel (sample DSA125) are shown in Fig. 3. No significant differences in infra-red spectra were found for samples prepared at other ammonium bicarbonate concentrations. Both spectra display a strong absorption peak near 1,100 cm^−1^ and a weaker feature near 800 cm^−1^ which can be assigned to the asymmetric and symmetric bending modes of Si–O–Si, respectively^20, 21^. The mode visible at ~ 960 cm^−1^, which can be assigned to Si–OH, indicates the presence of hydroxyl groups at the gel surface even after drying^20^. The spectrum from the wet gel shows intense absorption peaks due to the presence of water^22,23^—the broad strong band centered at 3,300 cm^−1^, the strong peak at 1638 cm^−1^ and the broad weak feature at ~ 2,100 cm^−1^. The band at 3,300 cm^−1^ can also still be observed in the dry aerogel, although at much lower intensity, indicating that water is still present and the aerogel is hydrophilic, consistent with TGA data presented in the Supplementary Material (Figure S4). The wet gel also displays additional features which are absent in the spectrum from the dried aerogel: two weak peaks are present at ~ 1,360 and ~ 1,455 cm^−1^ and are associated with the presence of the bicarbonate ion, HCO_3_^−^ and ammonium anion, NH_4_^+^, respectively^22^.

In aqueous solution NH_4_^+^ and HCO_3_^−^ are in equilibrium with NH_3(aq)_ and CO_2(aq)_^24^,

$$\begin{aligned}&{{{\rm NH}}_{4}^{+}}_{\left({\rm aq}\right)}+{{\rm OH}}_{\left({\rm aq}\right)}^{-}\leftrightarrow {{{\rm NH}}_{3}}_{\left({\rm aq}\right)}+{{\rm H}}_{2}{{\rm O}}_{\left({\rm l}\right)}\\ &{{{\rm HCO}}_{3}^{-}}_{\left({\rm aq}\right)}+{{\rm H}}_{\left({\rm aq}\right)}^{+}\leftrightarrow {{{\rm CO}}_{2}}_{\left({\rm aq}\right)}+{{\rm H}}_{2}{{\rm O}}_{\left({\rm l}\right)}\end{aligned}$$

so it is possible that during the heating process these solvated molecules are released as gas. Alternately if any solid ammonium bicarbonate forms it can decompose into NH_3(g)_, CO_2(g)_, and H_2_O_(l)_, with a decomposition rate strongly influenced by temperature. Its dissociation pressure at 25 °C is 7.85 kPa, but this rapidly increases to a value close to atmospheric pressure at a temperature of ~ 60 °C^24,25^.

As stated above, FT-IR spectra show the presence of both NH_4_^+^ and HCO_3_^−^ ions in the wet gel, but they disappear upon drying. The absence of any signal from bicarbonate in the dried aerogel indicates that the drying process leads to decomposition and elimination of this species, supporting the hypothesis for an aerogel drying mechanism analogous to that proposed by Han et al*.*^9^

**Figure 4:**
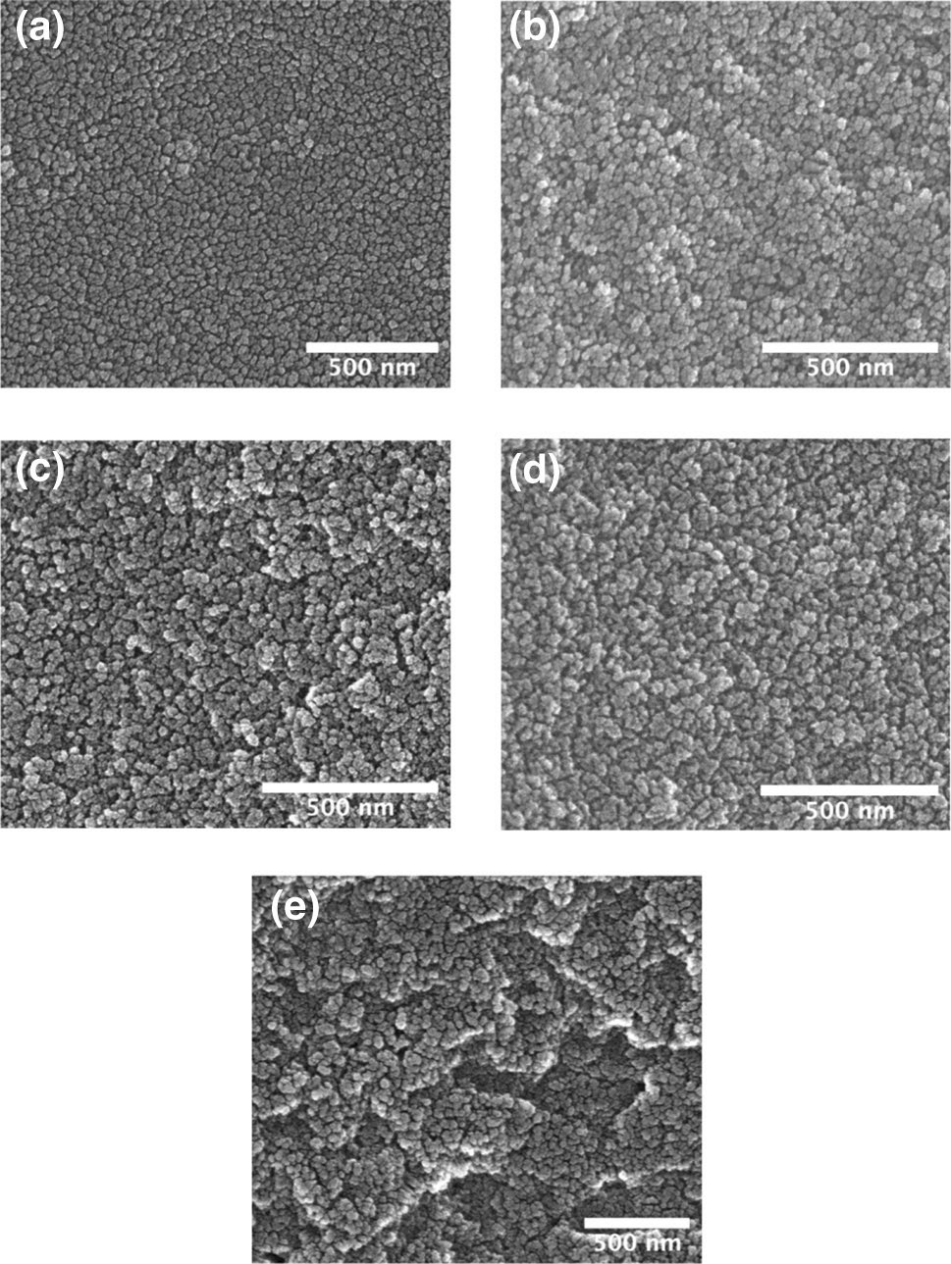
SEM images of undoped silica aerogels synthesized with different concentrations of ammonium bicarbonate: (**a**) 3 wt%, (**b**) 10 wt%, (**c**) 15 wt%, (**d**) 20 wt%, (**e**) 25 wt%.

SEM micrographs showing the microstructure of silica aerogels synthesized at different ammonium bicarbonate concentrations are shown in Fig. 4. All samples display a three-dimensional nanoporous structure. However, there is a coarsening of the structure with increasing ammonium bicarbonate concentration, reflected in an increase in the average feature size observed in the micrographs from ~ 260 to 550 nm^2^, which correlates well with the increase in specific surface area and pore size reported in Fig. 2. Such a correlation between the surface structure ‘particle’ size and pore size has also been observed in surface modified hydrophobic aerogels by Rao et al.^26^

**Figure 5:**
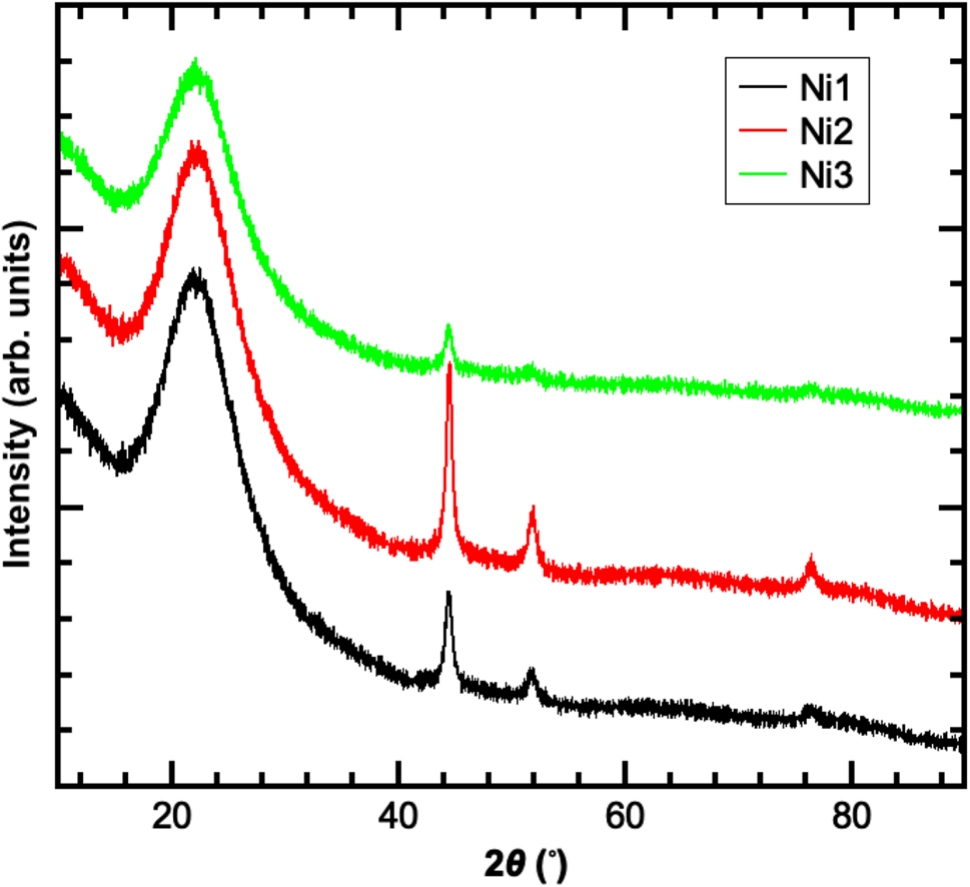
XRD 2*θ* scans obtained from nickel doped silica aerogels produced with varying concentrations of sodium hydroxide.

Nickel-doped silica aerogels were characterised with the same suite of techniques applied to the investigation of the undoped material. XRD scans for nickel-silica aerogel samples produced under different conditions are presented in Fig. 5. In addition to the broad peak between 2*θ* angles of 20° and 30° associated with amorphous silica which is also observed in the undoped silica aerogel (Figure S1), there are three sharp diffraction peaks located at 2*θ* values of 44.5°, 51.8° and 76.4° corresponding to the (111), (200) and (222) planes of crystalline face-centred cubic (fcc) nickel^14^ (JCPDS Card No. 04-0850) indicating the presence of the crystalline metal within the samples.

**Figure 6:**
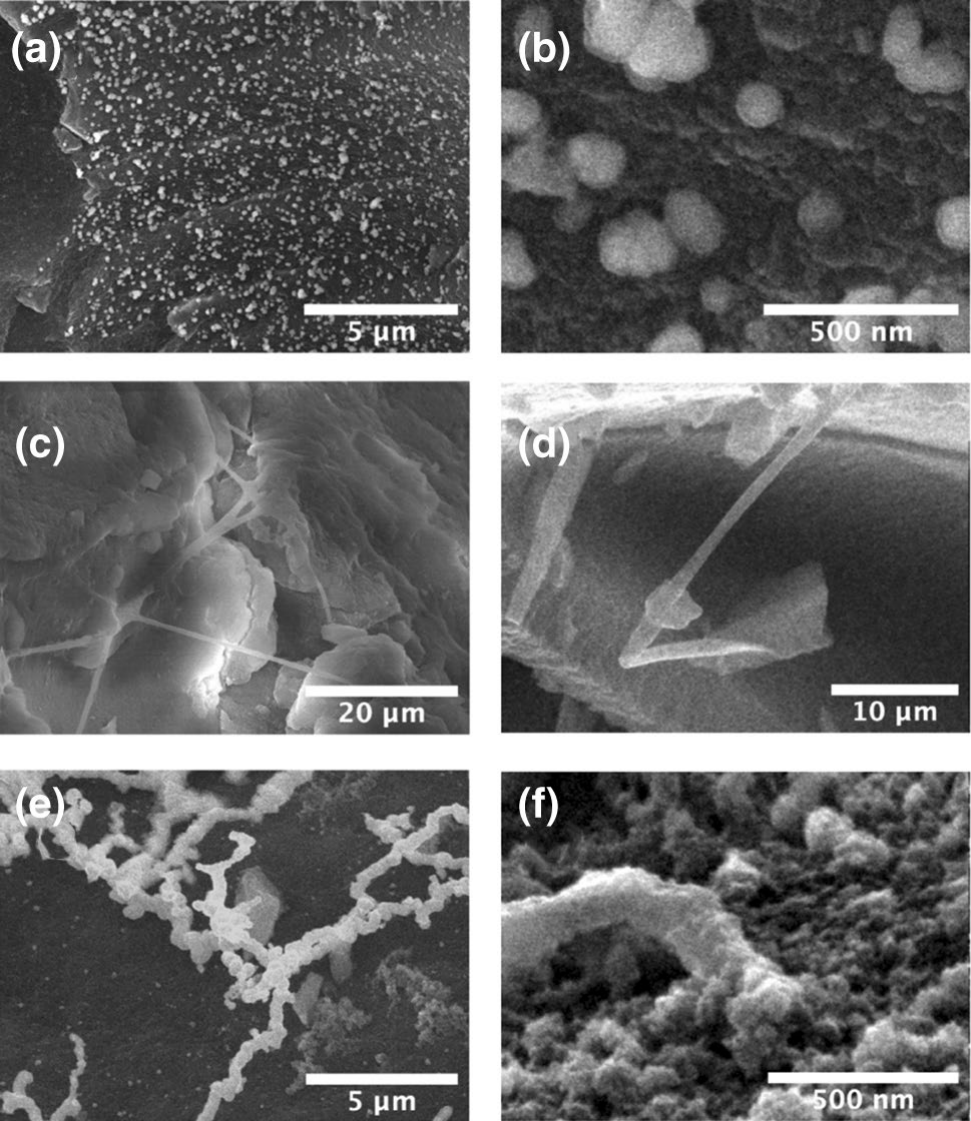
SEM images of nickel-silica aerogel synthesised with different concentrations of sodium hydroxide: (**a**,**b**) 0.5 M NaOH, (**c**,**d**) 1 M NaOH, (**e**,**f**) 2 M NaOH.

Insight into the morphology of the nickel within the samples can be found from SEM measurements on the fracture surfaces of the nickel doped silica aerogels, Fig. 6. When prepared with the lowest concentration of NaOH (0.5 M), spherical nanoparticles can be observed within the aerogel with average linear dimensions in the range between 50 and 200 nm. Energy dispersive X-ray analysis (EDX), presented in the Supplementary Material (Figures S5–S7), show a strong signal associated with nickel, confirming their composition.

A significant change in the nanoparticle morphology can be seen as the concentration of sodium hydroxide is increased. When 1 M NaOH is employed needle-like crystal structures with average diameters between 100 and 500 nm, rather than spherical nanoparticles are observed (Fig. 6c,d). Increasing the NaOH concentration still further, to 2 M, once more leads to a morphological change with the precipitate consisting of nanoparticles arranged in a chain-like morphology, with average particle diameters ranging between 100 and 500 nm (Fig. 6e,f). EDX once again demonstrates that the nanocrystal precipitates consist of nickel (Figure S7, Supplementary Material).

Nitrogen adsorption–desorption isotherms of the nickel nanoparticle doped silica aerogels (Figure S3, Supplementary Material) show the same type IV isotherm behaviour as the undoped aerogel indicating that the mesoporous structure remains unchanged. Neither the specific surface area nor the average pore diameters of the nickel-doped aerogel samples show any monotonic variation with sodium hydroxide concentration (Table 2), and pore volumes agree to within experimental uncertainty. The similarity between the average pore size of the doped samples and the undoped starting material indicates that the nickel does not significantly disrupt the structure of the aerogel. However, specific surface areas are reduced to between half and two-thirds that of the starting material suggesting that the nanoparticles may occlude some pores.

The agreement of pore structure measurements (surface area, pore volume and average pore size) of samples Ni1 and Ni2 within experimental uncertainty and the small variation from these in sample Ni3 indicates that the changes in morphology of the nickel nanoparticles is not related to any structural change of the aerogel, but rather to the nickel precipitation process. It is recognised that the role that NaOH plays in the formation of nickel nanostructures in the polyol process is as a catalyst, significantly increasing the rate of metal precipitation^17,27^ and that the rate of nucleation and growth of the metal nanoparticles can have significant impact on their morphology and dimensions. For example, starting with a nickel acetate tetrahydrate precursor rather than the NiCl_2_ used for the work reported here, Hinotsu et al*.*^28^ observed a significant changes in the NiNPs formed with increasing hydroxyl ion concentration with morphology changing from plate-like structures of micrometre dimensions to spherical particles a few hundred nanometres in diameter. Likewise, starting with a NiCl_2_ precursor, Eluri and Paul^27^ observed a decrease in NiNP size with increasing temperature and NaOH concentration, with the latter considered more significant. The variation in the nickel nanoparticle dimensions and morphology we observe with increasing NaOH concentration do not obviously follow these trends, indicating an interplay between the presence of the aerogel substrate and the rate of nickel precipitation. However, the conclusion can be drawn that the *changes* in the nanoparticle structure we observe with NaOH concentration are driven by rate of nickel precipitation, which in turns controls the rate of nanostructure nucleation and growth^27^.

## Conclusion

Silica aerogels with specific surface areas of up to 530 m^2^ g^−1^ have been produced by a novel ambient pressure drying technique modified from that reported by Han et al*.*^9^ in which a bicarbonate solution is fully decomposed with the application of heat, producing pore supporting gas within the body of the wet gel and preventing collapse during the drying process without leaving any residue. This has significant advantage over the original technique which leaves salt within the aerogel requiring washing for removal to avoid degradation of desirable properties such as ultra-low density. Precipitation of nickel within hydrophilic aerogels produced by this approach enables the growth of nanocrystalline metal within the body of the aerogel with morphology which varies considerably with growth conditions. Nickel nanocrystal morphologies ranging from spherical nanoparticles through needle-like crystals to wire-like nanoparticle chains are observed, controlled by the rate of nanostructure nucleation and growth. The dependence of nanoparticle catalytic activity on shape indicates that control over nickel nanocrystal growth can enable the tailoring of such doped aerogels for specific applications.

## Supplementary information

Supplementary Information.
