# Supplementary material for: Morphology control of nickel nanoparticles prepared in situ within silica aerogels produced by novel ambient pressure drying
Source: Sci Rep. 2020 Jul 16;10:11743. doi: 10.1038/s41598-020-68510-4 (PMC7366629; doi:10.1038/s41598-020-68510-4)
Supplement: Supplementary file 1 — Supplementary Information. [file 41598_2020_68510_MOESM1_ESM.docx]

**Supplementary Material for: Morphology control of nickel nanoparticles prepared in situ within silica aerogels produced by novel ambient pressure drying**

Jialu Lu, Jiabin Wang, Khalil T. Hassan, Alina Talmantaite, Zhengguang Xiao, Michael R.C. Hunt and Lidija Šiller

1. **XRD from undoped silica aerogel samples.**


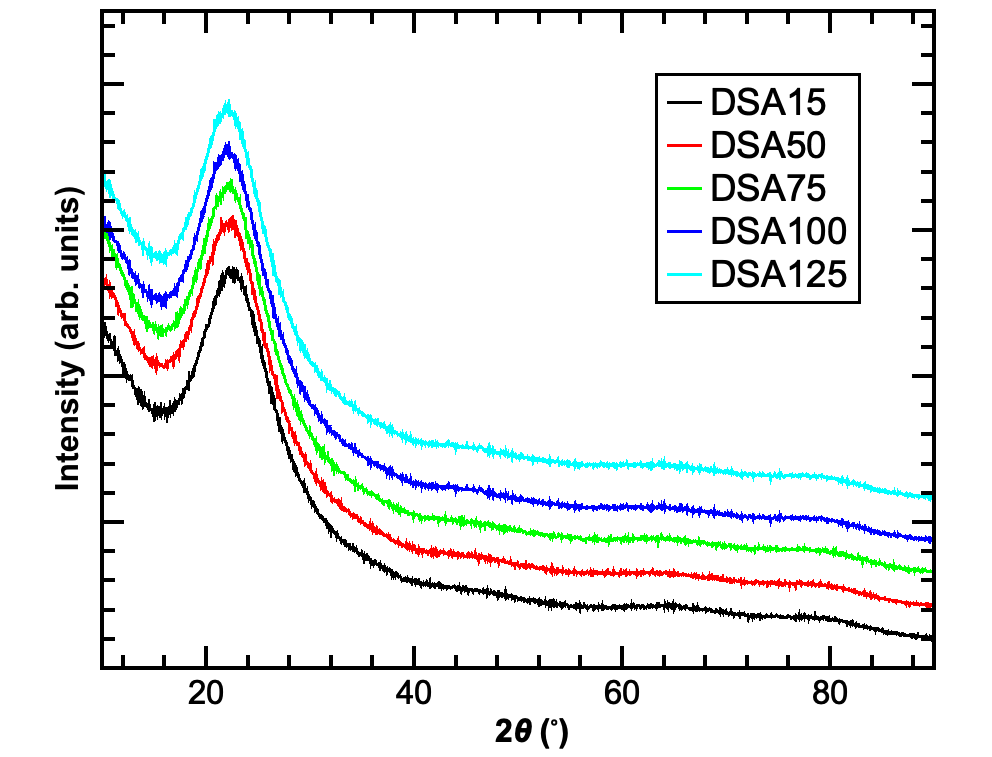


Fig. S1. XRD 2*θ* scans obtained from un-doped silica aerogels produced with varying concentrations of ammonium bicarbonate solution.

1. **N_2_ Adsorption-Desorption isotherms for undoped silica aerogel samples.**


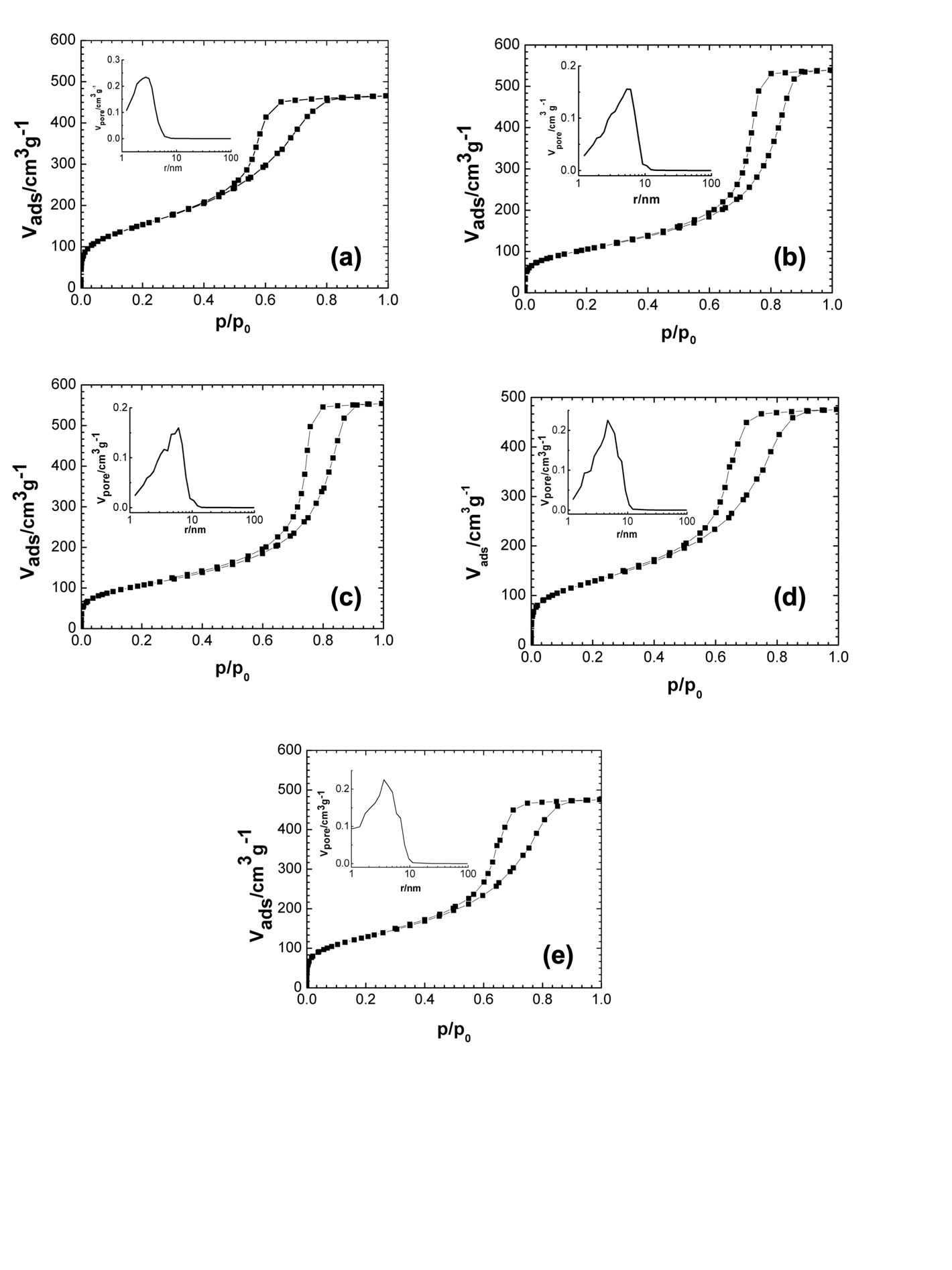


Fig. S2. N_2_ adsorption/desorption isotherms of silica aerogels prepared with different concentrations of ammonium bicarbonate: (a) 3 wt%, (b) 10 wt%, (c) 15 wt%, (d) 20 wt%, (e) 25 wt%. The insets show pore size distributions.

1. **N_2_ Adsorption-Desorption isotherms for Ni-doped silica aerogel samples.**


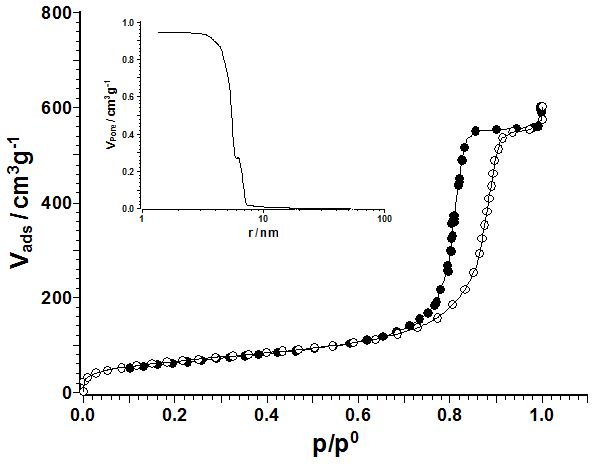

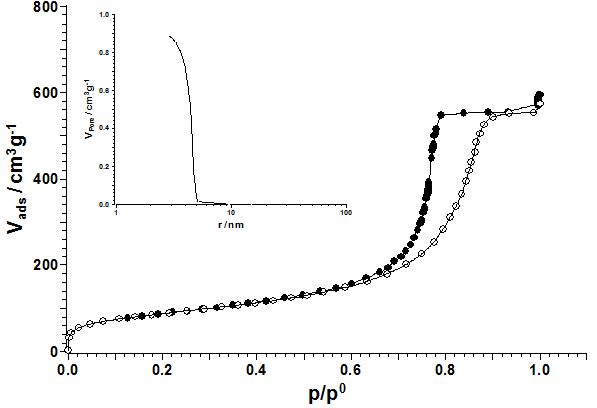

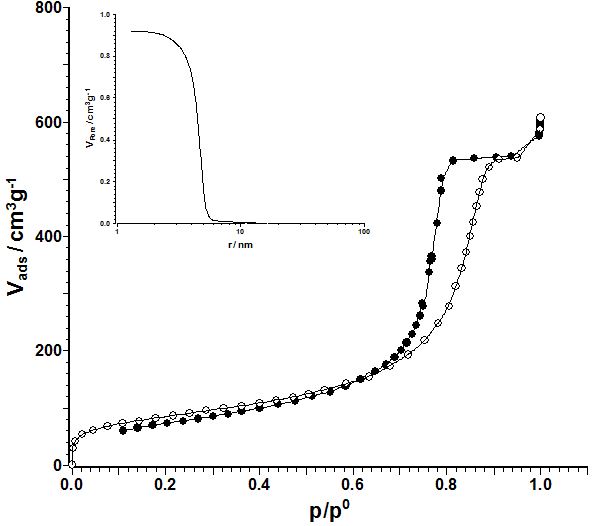


(b)

(c)

(a)

Fig. S3. N_2_ adsorption/desorption isotherms of nickel-doped silica aerogels prepared with different concentrations of sodium hydroxide: (a) 0.5M NaOH, (b) 1M NaOH, (c) 2M NaOH. The insets show pore size distributions.

1. **DTA data from silica aerogel samples.**


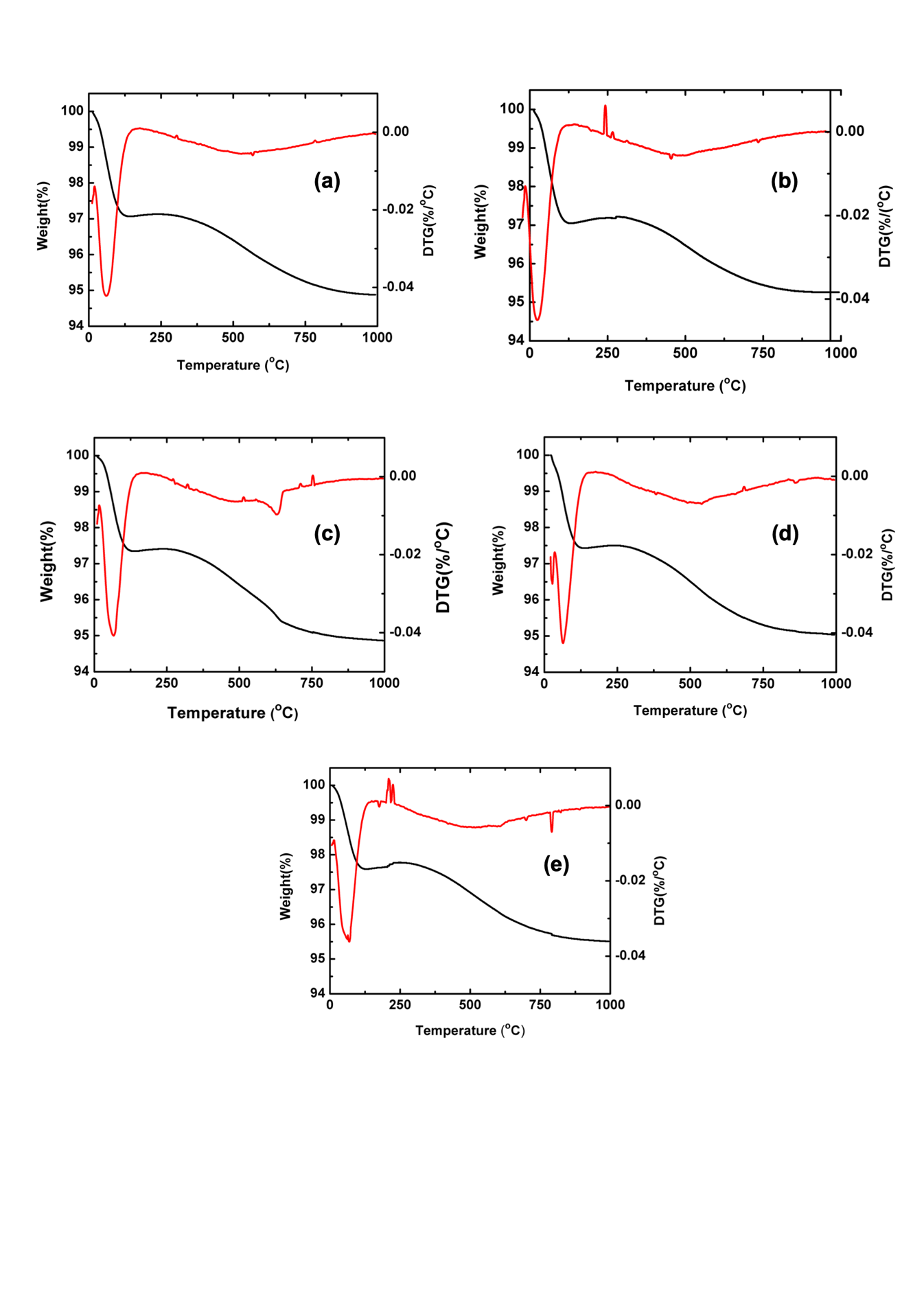


Fig S4. TGA-DTG from undoped silica aerogels synthesized with different concentrations of ammonium bicarbonate: (a) 3 wt%, (b) 10 wt%, (c) 15 wt%, (d) 20 wt%, (e) 25 wt%.

TGA data show a significant weight loss from room temperature to 120°C due to the evaporation of water from the silica aerogels. The weight losses for silica aerogels vary between 2.3% and 3%. Similar phenomena have also been observed for other hydrophilic silica aerogels ^1,2^_._  For all samples, there is another continuous weight loss from 200°C to 1000°C, likely to originate from continuous degradation of hydroxyl groups within the aerogel^3^. Derivative thermogravimetry (DTG) data are also plotted in Figure S4 to accentuate the rate of mass change.

1. **EDX Analysis of Ni-doped silica aerogels**


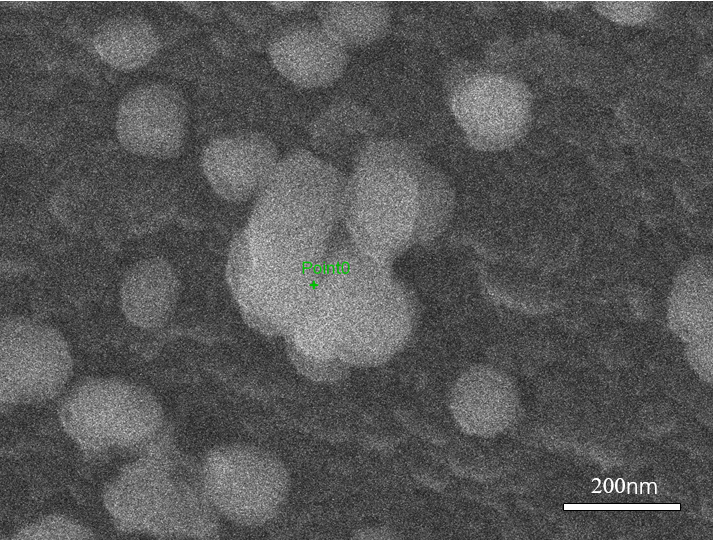


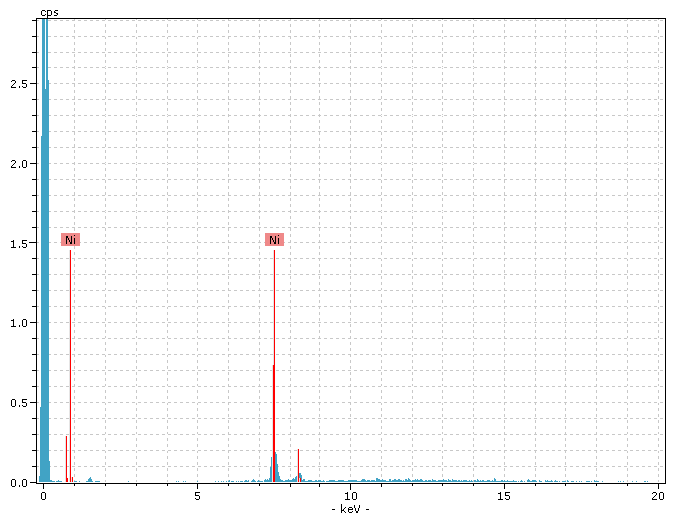


Fig. S5. SEM image (top) and corresponding EDX spectrum (bottom) of Ni-doped silica aerogel prepared with 0.5M NaOH. The EDX spectrum was acquired at the point marked in the SEM image. The spectrum demonstrates the presence of Ni in addition to Si from the aerogel skeleton.


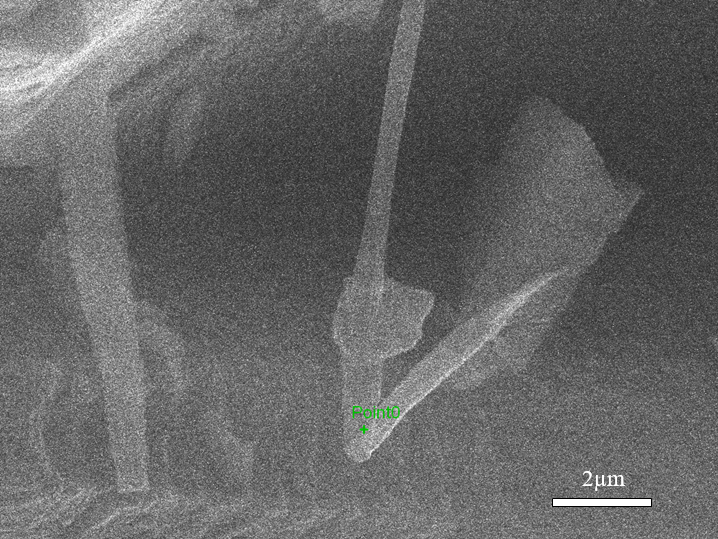


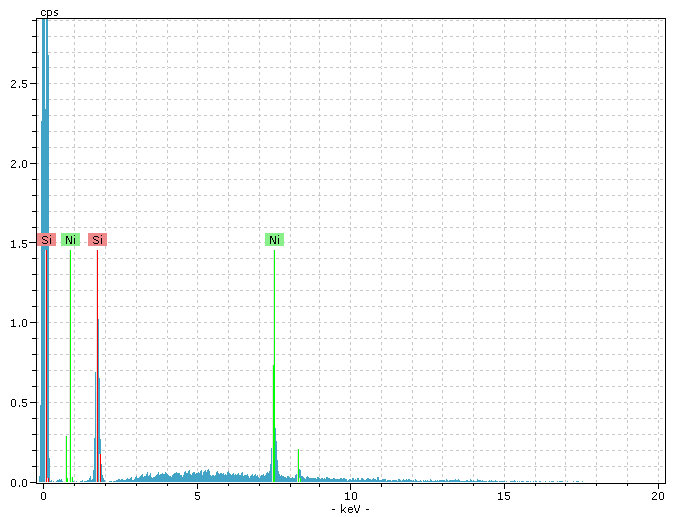


Fig. S6. SEM image (top) and corresponding EDX spectrum (bottom) of Ni-doped silica aerogel prepared with 1M NaOH. The EDX spectrum was acquired at the point marked in the SEM image. The spectrum demonstrates the presence of Ni in addition to Si from the aerogel skeleton.


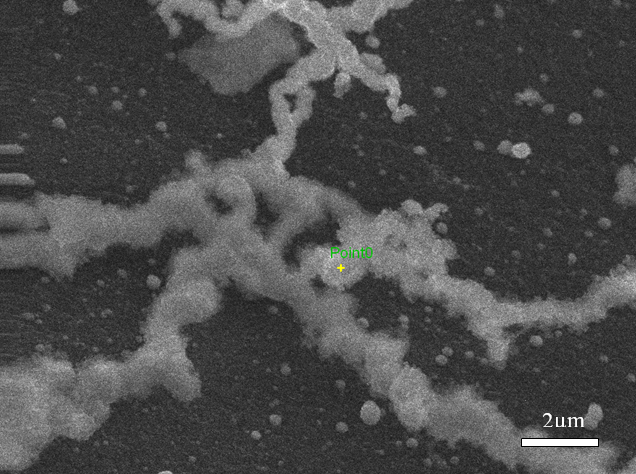


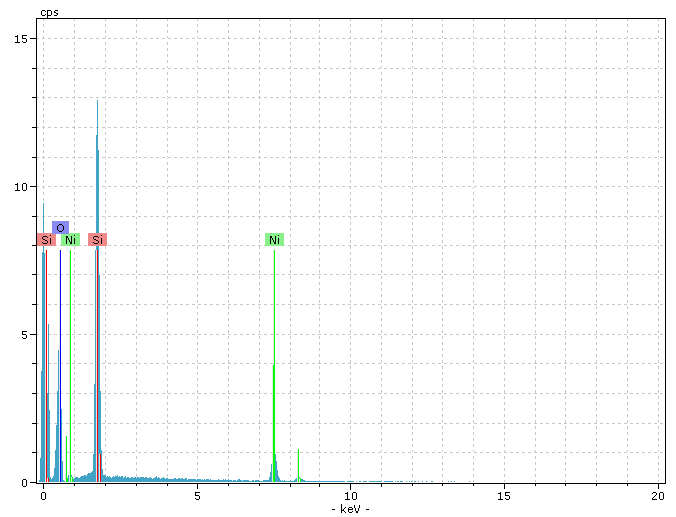


Fig. S7. SEM image (top) and corresponding EDX spectrum (bottom) of Ni-doped silica aerogel prepared with 2M NaOH. The EDX spectrum was acquired at the point marked in the SEM image. The spectrum demonstrates the presence of Ni in addition to Si and O from the aerogel skeleton.

1. **References**

1 Fazli, Y., Kulani, E., Khezri, K. & Alijani, H. PMMA-grafted silica aerogel nanoparticles via in situ SR&NI ATRP: Grafting through approach. *Micropor Mesopor Mat* **214**, 70-79. https://doi.org/10.1016/j.micromeso.2015.04.028 (2015).

2 Zhang, H., Li, C., Guo, J., Zang, L. & Luo, J. In Situ Synthesis of Poly(methyl methacrylate)/SiO_2_ Hybrid Nanocomposites via “Grafting Onto” Strategy Based on UV Irradiation in the Presence of Iron Aqueous Solution. *Journal of Nanomaterials* **2012**, 1-9. https://doi.org/10.1155/2012/217412 (2012).

3 Fazli, Y., Alijani, H. & Khezri, K. Styrene and Methyl Methacrylate Random Copolymerization via AGET ATRP: Incorporation of Hydrophobic Silica Aerogel Nanoparticles. *Advances in Polymer Technology* **35**, 260-268. https://doi.org/10.1002/adv.21549 (2016).
